# Of Mice, Men and Elephants: The Relation between Articular Cartilage Thickness and Body Mass

**DOI:** 10.1371/journal.pone.0057683

**Published:** 2013-02-21

**Authors:** Jos Malda, Janny C. de Grauw, Kim E. M. Benders, Marja J. L. Kik, Chris H. A. van de Lest, Laura B. Creemers, Wouter J. A. Dhert, P. René van Weeren

**Affiliations:** 1 Department of Orthopaedics, University Medical Center Utrecht, Utrecht, The Netherlands; 2 Institute of Health and Biomedical Innovation, Queensland University of Technology, Kelvin Grove, Queensland, Australia; 3 Department of Equine Sciences, Faculty of Veterinary Medicine, Utrecht University, Utrecht, The Netherlands; 4 Department of Pathobiology, Faculty of Veterinary Medicine, Utrecht University, Utrecht, The Netherlands; 5 Department of Biochemistry and Cell Biology, Faculty of Veterinary Medicine, Utrecht University, Utrecht, The Netherlands; Illinois Institute of Technology, United States Of Ameica

## Abstract

Mammalian articular cartilage serves diverse functions, including shock absorption, force transmission and enabling low-friction joint motion. These challenging requirements are met by the tissue’s thickness combined with its highly specific extracellular matrix, consisting of a glycosaminoglycan-interspersed collagen fiber network that provides a unique combination of resilience and high compressive and shear resistance. It is unknown how this critical tissue deals with the challenges posed by increases in body mass. For this study, osteochondral cores were harvested post-mortem from the central sites of both medial and lateral femoral condyles of 58 different mammalian species ranging from 25 g (mouse) to 4000 kg (African elephant). Joint size and cartilage thickness were measured and biochemical composition (glycosaminoclycan, collagen and DNA content) and collagen cross-links densities were analyzed. Here, we show that cartilage thickness at the femoral condyle in the mammalian species investigated varies between 90 µm and 3000 µm and bears a negative allometric relationship to body mass, unlike the isometric scaling of the skeleton. Cellular density (as determined by DNA content) decreases with increasing body mass, but gross biochemical composition is remarkably constant. This however need not affect life-long performance of the tissue in heavier mammals, due to relatively constant static compressive stresses, the zonal organization of the tissue and additional compensation by joint congruence, posture and activity pattern of larger mammals. These findings provide insight in the scaling of articular cartilage thickness with body weight, as well as in cartilage biochemical composition and cellularity across mammalian species. They underscore the need for the use of appropriate *in vivo* models in translational research aiming at human applications.

## Introduction

Articular cartilage is a heavily challenged tissue, as its main functions (shock absorption, force transmission and enabling low-friction movement of joints) require a combination of both great resilience and high compressive and shear resistance [1]. These demands are difficult to reconcile, but the tissue succeeds in doing so by the specific characteristics of its extracellular matrix (ECM) that consists of a glycosaminoglycan-interspersed collagen fiber network [2]. As articular cartilage is aneural, avascular and of low cellularity, its ECM is of relatively homogeneous composition. The downside, however, is that this constitution is thought to be the underlying cause of the very limited regenerative capacity of the tissue [3].

There is a huge difference in adult body mass amongst the currently living mammalian species. A mouse may weigh as little as 25 grams, whereas an African elephant easily reaches 4 tons, which represents 150,000-fold increase in body mass. The cube square law [4] stipulates that with increasing volume of a body, total mass increases with the third power of unit length, while the cross-sections of the supporting structures only increase with the second power, thus resulting in a linear increase in potential load (force per unit area) on these structures. The mammalian skeleton (y) generally scales proportionally [5] (isometrically; y  =  bx*^a^*; *a*  =  0.33) with body mass (x), and to compensate for the relatively higher loading of specific supporting structures, bone mass increases at certain sites[5], [6], [7], [8] and thus scales with positive allometry (*a*>0.33). However, the basic biological requirement for bone is to provide rigidity, which is more straightforward than the specific demands cartilage has to meet. Thus far, little is known about how articular cartilage deals with the challenges posed by increases in body mass [9]. The biochemical composition of the cartilage varies significantly over different topographical locations of the joint surface [10], [11], [12], and glycosaminoglycan (GAG) content appears to be dependent on local tissue loading [10], [13], [14]. While some significant differences in cartilage biochemical composition have been demonstrated between species [15], it is not known to what extent a similar mechanism would be necessary and may indeed exist to accommodate for the much larger differences in loading generated by the size differences between species.

Increases in thickness are likely to be limited by the avascular nature of cartilage. Previous studies in small groups of mammals, however, demonstrated that cartilage thickness does increase with increasing body mass [16], [17], [18]. Simon [16] found that cartilage thickness in 5 species of quadrupeds (mouse, rat, dog sheep, and cow) generally increased with body mass although marked variations were noted. Interestingly, Simon did not observe a consistent relationship between tissue thickness and the estimated compressive stress on the joint [16]. Stockwell [17] also showed that overall articular cartilage thickness is proportional to body mass in 8 mammalian species (mouse, rat, cat, rabbit, dog, sheep, man, and cow), although human cartilage was found to be relatively thicker. While these studies are helpful, they unfortunately comprised only a few species, were not fully conclusive, and failed to find evidence of a mechanism that may compensate for the more than proportional increase in potential loading that follows from the cube-square law.

We investigated the thickness and composition of the articular cartilage at the femoral condyle in 58 mammalian species with a wide variation in body mass. The hypotheses to be tested were that, (1) due to diffusional constraints [19], [20], cartilage thickness, unlike the dimensions of bones, cannot scale isometrically with increasing body mass and hence will be relatively thinner in larger animals; (2) a high cellularity of the articular cartilage could only be sustained in mammals with a low body mass; and (3) dramatic changes in extracellular matrix composition would not be required in view of the previously reported similar static compressive stresses in the articular cartilage of various species [16]. The results indeed show that cartilage thickness scales with negative allometry with body mass and that collagen and glycosaminoglycan content remain relatively constant over a wide body mass range.

## Materials and Methods

### Tissue harvest

Osteochondral cores were harvested post-mortem from the central sites of both the medial and lateral femoral condyles of different-sized mammals sent in for autopsy at the Department of Pathobiology, Faculty of Veterinary Medicine, Utrecht University, The Netherlands. Prior to harvest, animal species, age and body mass were recorded and macroscopic photographs of the joints were taken. Joints demonstrating macroscopic signs of cartilage degeneration, a microscopic Mankin score above 7 (see histology) or originating from skeletally immature animals were excluded. Human tissue samples were obtained from the Department of Pathology, University Medical Center Utrecht, The Netherlands, with approval of the local ethics committee and in line with the Dutch code of conduct “Proper Secondary Use of Human Tissue” as installed by the Federation of Biomedical Scientific Societies. In total, tissue was harvested (121 samples for histological and 84 for biochemical analysis) from mammals belonging to 58 different species (Table 1).

**Table 1:** Number of animals per species included in this study.

	*Species*	*Average body mass (kg)*	*Histology (n)*	*Biochemistry (n)*
1	Mouse (*Mus Musculus*)	0.025	5	
2	Pygmy marmoset (*Callithrix pygmaea*)	0.13	1	
3	Common marmoset (*Callithrix jacchus*)	0.3	1	1
4	Rat (*Rattus sp.)*	0.3	5	4
5	Cotton-top or Pinché tamarin *(Saguinus oedipus)*	0.34	1	1
6	Eurasian Red squirrel (*Sciurus vulgaris*)	0.4		1
7	Cape Ground squirrel (*Xerus inauris*)	0.65		1
8	Guinea pig *(Cavia porcellus)*	0.78	3	3
9	Potto (*Perodicticus potto*)	0.99	1	1
10	Ferret (*Mustela putorius furo*)	1.3	1	2
11	White-faced saki (*Pithecia pithecia*)	2	1	1
12	Ring-tailed lemur *(Lemur catta)*	2.2	1	2
13	Opossum (*Didelphis sp.*)	2.4	1	1
14	Oriental small-clawed otter (*Aonyx cinerea*)	2.81		1
15	Hare (*Lepus sp.*)	3.1	2	4
16	Rabbit *(Oryctolagus cuniculus)*	3.7	6	7
17	South American coati (*Nasua Nasua)*	5.1	2	1
18	European otter (*Lutra lutra*)	6.5	1	1
19	Linnaeus's two-toed sloth (*Choloepus didactylus*)	6.5	1	1
20	Black Mangabey (*Lophocebus albigena*)	7	1	1
21	Vervet monkey *(Chlorocebus pygerythrus)*	7.7	2	1
22	Southern or Chilean Pudú (*Pudu puda*)	7.8	2	2
23	Woolly Monkey *(Lagothrix lagotricha)*	8.4	1	1
24	Barbary macaque (*Macaca sylvanus*)	8.5	2	2
25	Badger (*Meles meles*)	10	2	2
26	Dikdik (*Madoqua kirkii*)	10	1	
27	Beagle dog (*Canis sp.*)	12	4	2
28	Tammar wallaby (*Macropus eugenii*)	12.5	2	1
29	Hamadryas baboon (*Papio hamadryas*)	15.8	3	3
30	Indian crested porcupine (*Hystrix indica*)	16	1	1
31	Thomson’s gazelle (*Eudorcas thomsoni*)	18	4	1
32	Roe deer (*Capreolus capreolus*)	19.2	5	2
33	Capybara *(Hydrochoerus hydrochaeris)*	22	1	1
34	Dutch milk goat (*Capri hircus*)	25	1	
35	West African dwarf goat (*Capri sp.*)	29	1	1
36	Cheetah (*Acinonyx jubatus*)	39.5	4	1
37	Impala (*Aepyceros melampus*)	41	2	2
38	Red Kangaroo (*Macropus rufus*)	52.5	2	1
39	Human (*Homo Sapiens*)	68.3	10	2
40	Fallow deer (*Dama dama*)	70	1	1
41	Gorilla (*Troglodytes gorilla*)	74		1
42	Siberian tiger (*Panthera tigris*)	80	1	1
43	Reindeer (*Rangifer tarandus*)	125	1	
44	Lion *(Panthera leo))*	148	1	
45	Horse (mini-shetland) (*Equus sp.*)	150	1	
46	Kudu (*Tragelaphus strepsiceros*)	150	1	
47	Llama (*Lama Glama*)	160	1	
48	Polar bear (*Ursus Maritimus*)	175	1	1
49	South American tapir (*Tapirus terrestris*)	250	1	1
50	European moose (*Alces alces alces*)	343	1	1
51	Watoessi (*Bos Taurus Taurus watussi*)	350	1	
52	Dairy cow (*Bovinae*)	450	2	
53	Giraffe (*Giraffa camelopardalis*)	555	3	1
54	Horse (*Equus ferus caballus*)	557	15	13
55	Banteng (*Bos javanicus*)	600	1	1
56	White rhinoceros (*Ceratotherium simum*)	1550	2	2
57	Asian elephant (*Elaphus maximus*)	3350	2	1
58	African Elephant *(Loxodonta africanus)*	4000	1	
	**Total**		**121**	**84**

### Histology

Osteochondral tissue samples for histology were decalcified using Luthra solution (3.2% 11 M HCl, 10% formic acid in distilled water), dehydrated, cleared in xylene, embedded in paraffin and cut to yield 5 µm sections. Sections were either stained with hematoxylin and eosin for image analysis or with hematoxylin, fast green and safranin-O for measurement of cartilage thickness from the surface down to the chondro-osseous junction and for osteoarthritic grading using the Mankin score [21]. Digital images were analyzed using cell^ ˆ^F software (Olympus, USA). The average thickness of the articular cartilage of each sample was determined by averaging 4 measurements per image at different locations.

### Glycosaminoglycan and DNA content

Cartilage samples for biochemical analyses were digested overnight at 60°C in 20 µL papain solution (0.01 M cysteine, 250 µg/ml papain, 0.2 M NaH_2_PO_4_ and 0.01 M EDTA.2H_2_O) per mg cartilage tissue. Glycosaminoglycan (GAG) content of the digests was determined spectrophotometrically after reaction with dimethylmethylene blue reagent (DMMB, Sigma-Aldrich, USA) [22]. DNA content was determined using the Picogreen DNA assay [23] (Invitrogen, P7589) in accordance with the manufacturer’s instructions.

### Collagen content

Hydroxyproline content (as a measure of collagen content) and collagen cross-links were analyzed by HPLC-MS/MS using multiple reaction monitoring (MRM) as previously described [24]. Briefly, aliquots of digested cartilage samples were hydrolyzed (110°C, 18–20h) in 6 M HCl. Homo-arginine was added to the hydrolyzed samples as an internal standard. Samples were vacuum-dried and dissolved in 30% methanol containing 0.2% heptafluor buteric acid (HFBA). After centrifugation at 13,000 *g* for 10 min, the supernatants were analyzed with HPLC-MS/MS, using an API3000 mass-spectrometer (Applied Biosystems/MDS Sciex, Foster City, CA) at a source temperature of 300°C and a spray voltage of 4.5 kV. Amino acids were separated on a Synergi MAX-RP 80A (250×3 mm, 4 µm) column (Phenomenex Inc., Torrance, CA) at a flow rate of 400 µL/min, using a gradient from 0.2% HFBA in MilliQwater (Millipore, Billerica, MA) to 100% methanol (Biosolve, Valkenswaard, The Netherlands).

### Statistics

Statistical comparison of the medial and lateral cartilage thicknesses was conducted using a paired one-sample Student’s t-test on the ratios. For correlations between body mass and cartilage thickness, a regression analysis using a power curve fit was performed. Statistical comparison of the obtained power coefficient with the theoretical coefficient of 0.33 (isometric scaling) was performed using a one-sample T-test. Significance of both tests was assumed at p<0.05.

## Results

**Figure 1:**
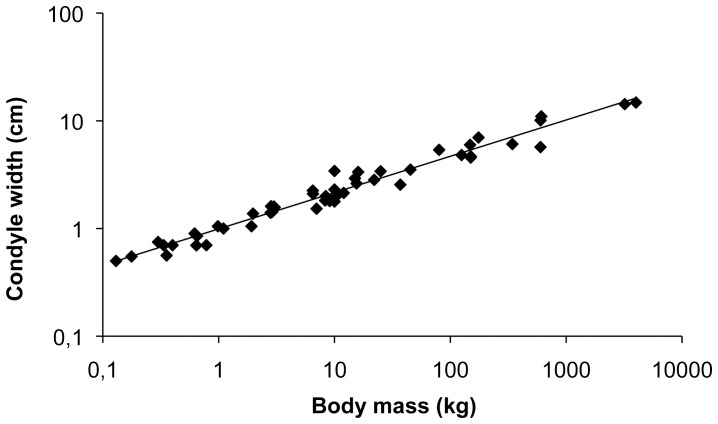
Scaling of the knee joint. The total average width of the articulating lateral and medial condyles per species follows an isometric relationship with body mass (*a* = 0.337, R^2^ = 0.96), illustrating the isometric scaling of the entire skeleton. Image shows the lateral and medial condyles of a cheetah.

**Figure 2:**
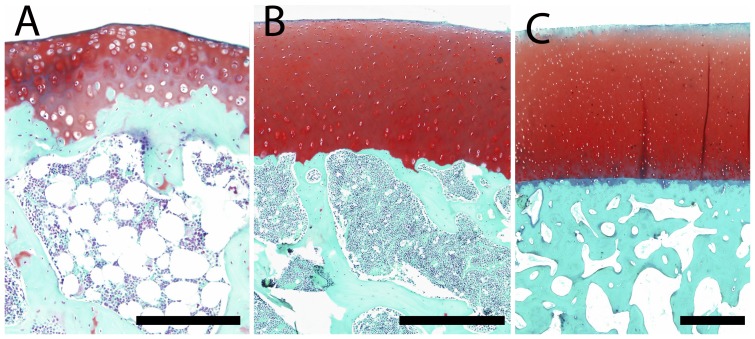
Safranin-O staining (stains GAGs red) of osteochondral tissue of the (A) rat, (B) barbary macaque and (C) white rhinoceros. Scale bars indicate (A) 200 µm, (B) 400 µm, and (C) 1000 µm.

The total width of the lateral and medial condyles was analyzed (Figure 1) as a measure of joint size in the 58 different species of mammals evaluated (Table 1). We found an increase in total condyle size with body mass that scaled according to an isometric relation (*a = *0.337, Figure 1), in line with previous observations on the scaling of the mammalian skeleton. Histological analysis revealed a relatively higher bone density of the subchondral bone in larger species in our study (Figure 2).

Within the cartilage tissue of all species, a decreased intensity of safranin O staining was observed within the superficial layers compared to the deeper layers, indicative of lower glycosaminoglycan content in the upper tissue regions (Figure 2).

**Figure 3:**
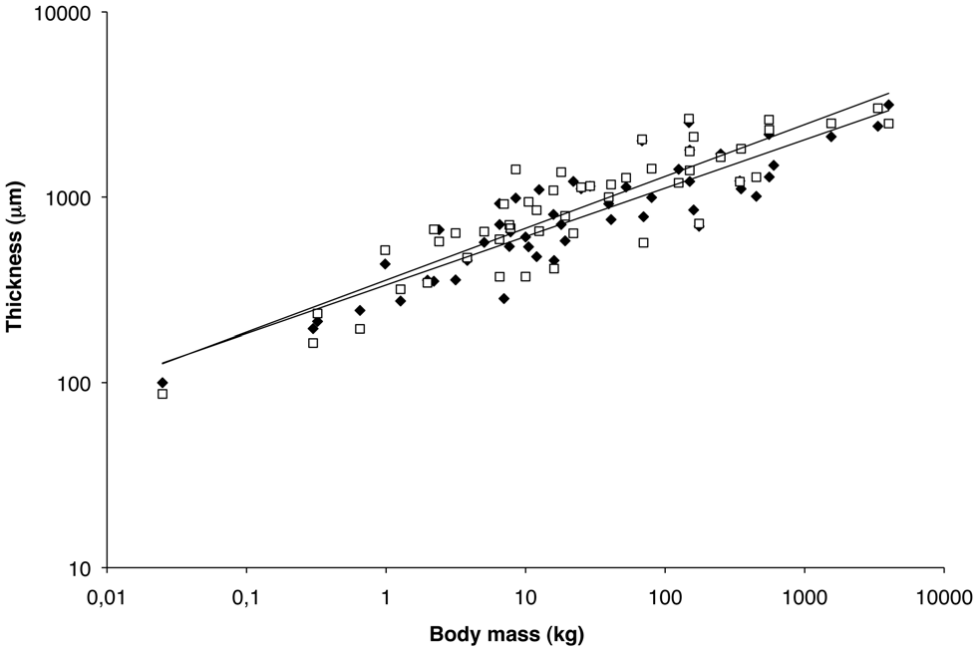
Average mammalian articular cartilage thickness per species at the center of lateral (black diamonds) and medial (open squares) condyles varies allometrically with body mass (*a* = 0.262 and *a* = 0.280, respectively).

We found that the thickness of the calcified plus non-calcified cartilage layer on the summits of the lateral and medial femoral condyles varied widely between species (Figure 3), ranging from about 90 µm in the mouse to 2,000 µm in humans and approximately 3,000 µm in the Asian elephant (Figure 3, Table 2). Moreover, cartilage thickness was (on average per species) significantly greater at the medial than at the lateral condyle (15%, p = 0.004).

**Table 2:** Cartilage thickness at the lateral en femoral condyles of selected species.

Species	(n)	Thickness Lateral ±SD ( µm)	Thickness Medial ±SD ( µm)
Mouse (Mus Musculus)	5	99±32	87±13
Rat (Rattus sp.)	5	213±29	235±46
Rabbit (Oryctolagus cuniculus)	6	455±119	470±139
Vervet monkey (Chlorocebus pygerythrus)	2	540±142	707±48
Beagle dog (Canis sp.)	4	476±146	849±184
Hamadryas baboon (Papio hamadryas)	3	805±85	1087±145
Cheetah (Acinonyx jubatus)	4	919±152	999±297
Human (Homo Sapiens)	10	2014±512	2050±780
Horse (Equus ferus caballus)	15	1283±205	2309±726
White rhinoceros (Ceratotherium simum)	2	2119*	2502±192
Asian elephant (Elaphus maximus)	2	2413±101	3021±335

* = only one sample was available.

There was a direct relationship between cartilage thickness and body mass, but our data reveal that cartilage thickness increased less than would be expected based on isometric scaling of the skeleton (as illustrated in Figure 1), and consequently bore a negative allometric relationship to body mass over the range 25 g (mouse) – 4,000 kg (African elephant) for both the lateral (*a* = 0.262; R^2^ = 0.80, p<0.001) and medial (*a* = 0.280; R^2^ = 0.79, p<0.001) condyles (Figure 3). The obtained power coefficients (*a*) were significantly different from the theoretical coefficient of 0.33 for both lateral (p<0.001) and medial (p = 0.01) sites.

**Figure 4:**
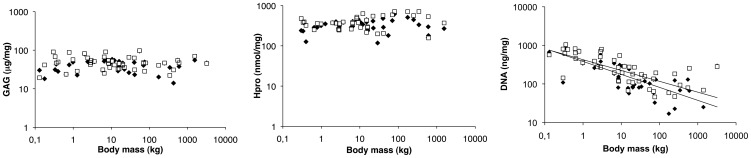
Average (A) glycosaminoglycan (GAG) and (B) hydroxyproline (Hpro) content of the articular cartilage per species is independent of body mass, whilst an inverse relation was observed for (C) DNA at the lateral (black diamonds, *a* = -0.327) and medial (open squares, *a* = -0.282) condyles.

The average overall GAG content across species (lateral: 47±14 µg per mg, medial: 49±15 µg per mg cartilage) appeared not to be related to body mass (Figure 4A). In addition, hydroxyproline content, as a measure of collagen content, (lateral: 350±154 nmol hydroxyproline per mg, medial: 419±180 nmol hydroxyproline per mg) was also independent of body mass across different species (Figure 4B). In contrast, an inverse relationship between DNA content and body mass was observed (lateral: R^2^ = 0.50 and medial: R^2^ = 0.51) (Figure 4C), resulting in a rapid decrease in DNA content with increasing body mass, particularly in the 25 g–10 kg range.

**Figure 5:**
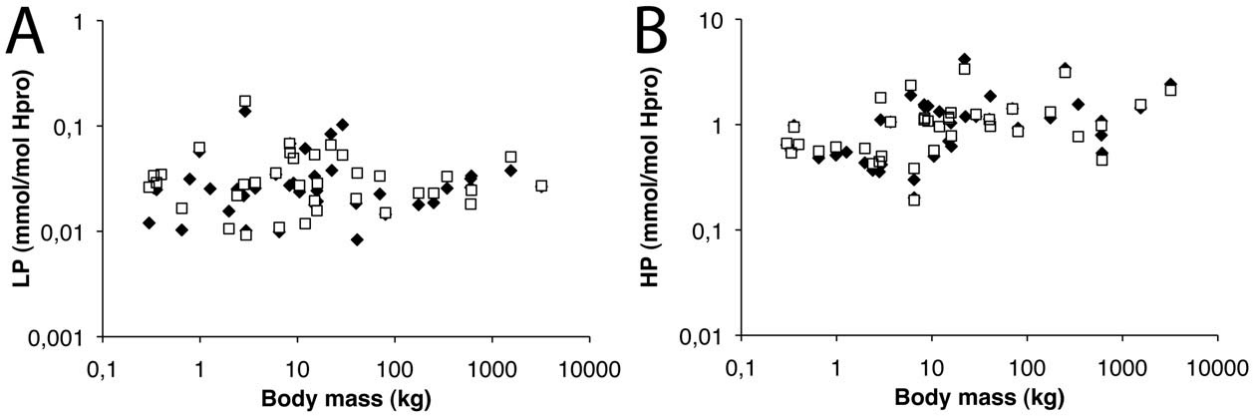
Average collagen cross-link content as a function of body mass. (A) Lysyl-pyridinoline (LP) and (B) hydroxylsyl-pyridinoline (HP) cross-links are independent of body mass at the lateral (black diamonds) and medial (open squares) condyles.

Since structural features of the collagen network might also influence the mechanical properties of the tissue, collagen cross-links were analyzed as well. However, no significant correlation between lysyl-pyridinoline (LP) or hydroxylsyl-pyridinoline (HP) cross-link density and body mass was found (Figure 5).

## Discussion

The present study shows for the first time that cartilage thickness at the femoral condyle bears a negative allometric relationship body mass, unlike the size of the mammalian skeleton that generally scales proportionally (isometrically) with body mass [5], [6], [7], [8]. In addition, we show that cellular density (as determined by DNA content) decreases with increasing body mass particularly in the lower end of the mass spectrum, but that gross biochemical composition is remarkably constant over a wide range of mammalian body mass.

The condylar cartilage thicknesses reported here are in line with the outcomes of earlier studies investigating cartilage thicknesses in small groups of animals of different species [16], [17], [18]. Moreover, the average greater thickness of the medial compared to the lateral condyle is also in line with previous reports on a number of different species including the horse [25], cow [26], sheep [27] and rabbit [28]. Cartilage thickness scaled according to a negative allometric relationship with body mass; *i.e*., based on the thickness observed in small mammals and assuming proportional scaling, one would have expected a considerably greater tissue thickness (approximately 4,500–6,000 µm) than the actual observed value (3,000 µm) for the African elephant. This lower-than-expected increase in tissue thickness may be related to diffusional constraints, as adult articular cartilage lacks vascularization [20], [29]. Interestingly, recent research on fossilized material of the largest land creatures that ever lived, the dinosaurs, revealed traces of vascularization to potentially sustain the substantially thicker articular cartilage [30]. In contrast to our findings, previous investigations [16], [17], [26] have suggested a positive allometric relationship between articular cartilage thickness and body mass. These studies were however performed on only a small number (5–8) of mammalian species of less than 300 kg, analyzed the maximum cartilage thickness in the joint and included skeletally immature animals [16], [17], [26]. These factors likely explain the overestimation of cartilage thickness in the larger species in these studies.

Besides variation in thickness, the mechanical characteristics of articular cartilage are determined by the interplay of its three main biochemical constituents: collagen, proteoglycans and water. Although some species differences in biochemical composition of the articular cartilage have previously been demonstrated[15], we found that gross biochemical composition is remarkably constant. It should be noted however that the DMMB assay [22] we employed is a rather crude technique for GAG quantitation that for example does not discriminate between keratan sulphate and chondrotin sulphate [31]. The ratio of these components in the cartilage may significantly affect the overall fixed charge density [31], which in turn will affect the mechanical characteristics of the tissue [32]. Nevertheless, our results indicate a certain immutability of cartilage ECM with respect to gross composition, as both collagen content and the abundance of pyridinoline cross-links that heavily influence mechanical properties, were likewise found to be relatively stable over a wide range of mammalian body mass.

Cartilage DNA content, as a measure of cellular density, decreases with increasing body mass. This observation is consistent with the finding that cell density in thinner cartilages is considerably higher [17], although in the current study potential species-specific differences in DNA content per cell were not taken into account. The relatively high DNA content in mouse and rat cartilage is not a specific feature of rodent cartilage, as the cartilage of the Capybara (the largest extant rodent in the world), showed considerably lower DNA content, which is in the range of other similarly-sized mammals. The high DNA content of the thinner cartilages could also be related to the high cell content in the superficial zone of the tissue [17], [25], [33], [34], which likely contributes relatively more to total tissue thickness in thin cartilage than in thicker cartilage. Regardless, the relatively high DNA content in the lighter species is indicative of a higher cellularity of thinner cartilage (as supported by histological evaluation of tissue cell density). This may impact (positively) on the regenerative capacity of cartilage in these smaller animals and underscores the need for the use of appropriate *in vivo* models [35], [36], which approximate the human situation, when evaluating experimental approaches for cartilage repair.

An increase in mammalian body mass will require adaptations of the musculoskeletal system to accommodate for higher loading. Alterations in tissue dimensions and/or composition constitute a logical response to such changes. Indeed, articular cartilage biochemical composition (and with it biomechanical characteristics) have been shown to be both location and age dependent [37], [38], [39], which may explain the higher variation of the biochemical data in comparison to the joint sized in our study.

When gross ECM composition is remarkably stable over a large range of species and body mass, differences may exist at a more detailed (structural / molecular) level. These may explain previously reported interspecies differences in mechanical properties [32]. Although cartilage is a relatively homogeneous tissue, distinct zones, each with their own specific compressive properties, biochemical composition and structural organization, can be distinguished from the articular surface down to the cartilage-bone interface. For example, the superficial zone is known to exhibit larger strain [40] and to have lower GAG content [25] compared to deeper zones (in line with our histological safranin O stainings). Moreover, the superficial zone contains a number of specific extracellular matrix components, including lubricin (proteoglycan-4) [41] and clusterin [42] that are not found in the deeper zones. In addition, the chondroitin sulphate sulphation motifs and the ratio of chondroitin sulphate to keratan sulphate also vary with depth [31], [43]. Collagen content is relatively stable throughout the depth of the tissue [25], but collagen fibril orientation is notably depth-dependent [44]. These depth-dependent differences clearly have implications for the overall mechanical characteristics of tissue with a specific thickness and may hence contribute to the adaptation to higher loads. Consequently, the potential variation in depth-dependent biochemical properties of the cartilage over a range of species and body masses warrants further investigation.

The limited increase in thickness of cartilage and its biochemical constancy are probably largely compensated for, as supported by the fact that static compressive stresses in the joint cartilage among various species are within one order of magnitude and are unrelated to cartilage thickness [16]. Moreover, compression of the tissue is radially confined and shear forces are further resisted by bonding with the subchondral bone and periarticular structures. This, together with the increase in joint surface area in the larger species and accompanying changes in joint alignment, posture and activity pattern (which are related to body mass [9]), might be sufficient to compensate for the additional loading. However, whether the less-than-proportional increase of articular cartilage thickness in larger mammals contributes to a greater susceptibility to degenerative joint disorders in these animals remains unclear and could be an interesting area of future investigation.

## Conclusion

Articular cartilage thickness scales according to negative allometry, and, as a result, cartilage is relatively thinner in larger animals. This is potentially due to diffusional constraints, as is illustrated by the presence of high cell densities only in thin cartilages. However, gross biochemical composition is remarkably constant over a wide range of body mass, which, together with the negative allometric scaling of thickness, theoretically leads to a decrease in biomechanical resistance with increasing body weight. However, an isometric increase in thickness may not be required for life-long performance, in light of relatively constant static compressive stresses on the tissue perhaps facilitated by additional compensatory factors like congruence, posture and activity pattern of the animal.
